# Silicon Carbide-Based Anodes for Lithium-Ion Batteries: A Green View

**DOI:** 10.1021/acsomega.5c07155

**Published:** 2025-11-25

**Authors:** Ana Isabela Pianowski Salussoglia, João Paulo de Mesquita, Marcio Cesar Pereira, Rafael Vicentini, Gustavo Doubek, Hudson Zanin

**Affiliations:** † Advanced Energy Storage Division, Center for Innovation on New Energies, 28132 University of Campinas, School of Electrical Engineering and Computer, Campinas, São Paulo 13083-852, Brazil; ‡ Department of Chemistry, 74380 Federal University of Jequitinhonha and Mucuri Valleys, Diamantina, Minas Gerais 39100-000, Brazil; § Institute of Science, Engineering, and Technology (ICET), Federal University of Jequitinhonha and Mucuri Valleys (UFVJM), Campus Mucuri, Teófilo Otoni, Minas Gerais 39803-371, Brazil

## Abstract

Silicon carbide (SiC) has evolved from an inert structure to a potential candidate for lithium storage, offering an attractive alternative to graphite and silicon anodes. This review unifies experimental insights and theoretical predictions to elucidate the mechanisms that govern lithiation, conversion, alloying, and interfacial storage while clarifying the roles of polytype, nanostructure, and defect chemistry. Emphasis is placed on sustainable synthesis routes that valorize biomass and industrial residues, alongside aqueous and bioderived binders that couple interfacial stability with green processing. By benchmarking bare SiC and heterostructured composites against state-of-the-art anodes, we expose unresolved controversies, highlight design principles for nanoscale activation, and identify pathways toward scalable low-carbon fabrication. This review establishes SiC as more than a mechanistic curiosity, positioning it as a viable and sustainable anode candidate, and provides a critical roadmap for accelerating the rational design of next-generation lithium-ion batteries.

## Graphical abstract

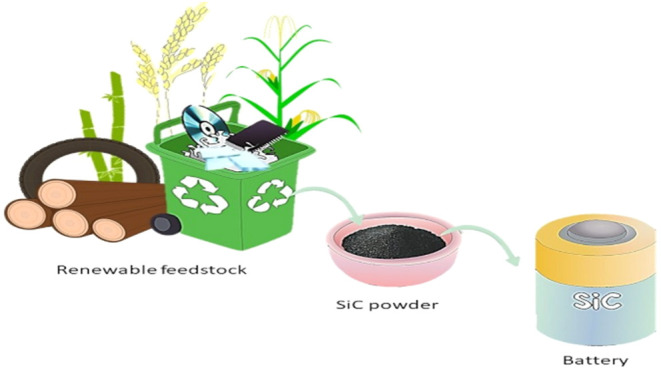

## 1 Introduction

Anodes remain one of the most critical bottlenecks in advancing lithium-ion batteries (LIBs), as the search for high-capacity and sustainable alternatives to graphite continues to face unresolved trade-offs. Batteries are central to global sustainability efforts, enabling reductions in greenhouse gas emissions and supporting the transition away from fossil fuels.^1^ Political initiatives and environmental policies aimed at phasing out fossil-fuel-powered vehicles further accelerate the demand for high-performance and environmentally benign energy-storage technologies.^2,3^ Among competing chemistries, LIBs dominate due to their high energy density and versatile applications ranging from electric vehicles to grid-scale storage.^4^ In these systems, lithium ions shuttle between the anode and cathode through an electrolyte (Figure 1), while the separator ensures selective transport and prevents internal short circuits.^5,6^

**Figure 1:**
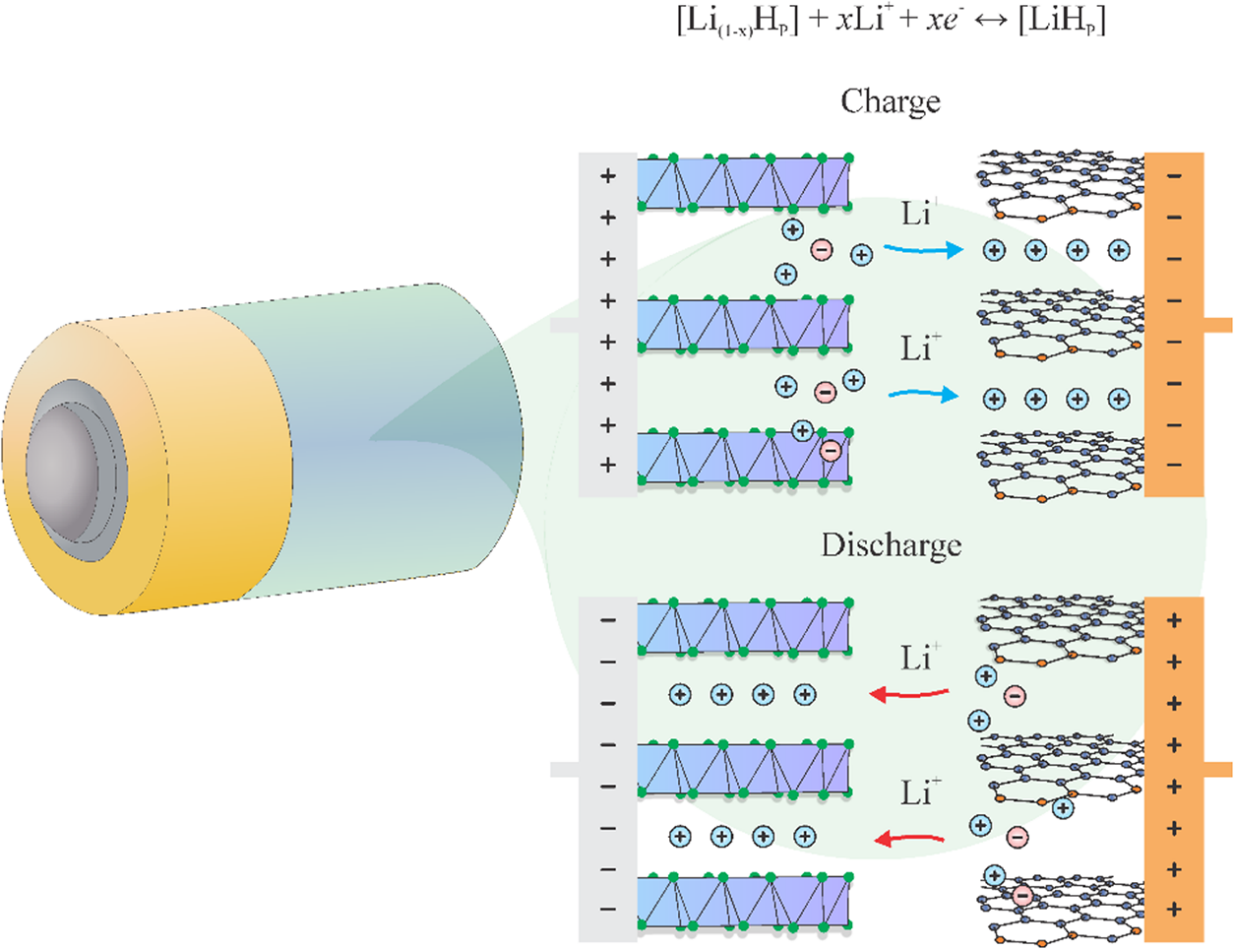
Schematic illustration of the LIB working.

The performance of LIBs is strongly dictated by the choice of anode material.^7–11^ Graphite, the commercial standard, benefits from low cost and stable cycling derived from its layered structure, which accommodates reversible Li intercalation with minimal volume change. However, its theoretical capacity is limited to only 372 mAh g^–1^,^12,13^ which constrains further energy-density improvements. However, the market demands higher power and energy densities, especially for electric vehicles, necessitating the development of advanced anode materials.^14^ Silicon has attracted widespread interest as a potential replacement, owing to its ultrahigh theoretical capacity of ∼4200 mAh g^–1^, low delithiation potential,^15–17^ and natural abundance.^18^ However, severe drawbacks, including extreme volume expansion during lithiation, poor intrinsic electrical conductivity, and unstable solid-electrolyte interphase (SEI), lead to rapid capacity fade and hinder commercialization.^19–24^ Considerable efforts have been devoted to mitigating these limitations through nanostructuring, composites, and surface modifications.^15–17,25^

Within this context, silicon carbide (SiC) has emerged as a promising alternative anode material. SiC offers a distinctive combination of mechanical robustness, thermal and chemical stability, and tunable electronic properties that, in principle, could reconcile the trade-offs observed in Si and graphite. Recent advances in synthesis, particularly carbothermal and magnesiothermic reductions, have enabled the fabrication of diverse morphologies, including nanofibers, nanowires, nanorods, spheres, and nanosheets, which facilitate lithium-ion diffusion and stabilize the SEI.^26^ However, significant challenges remain because the intrinsically low electrical conductivity of SiC requires conductive modification,^27,28^ and the high processing temperatures raise concerns about scalability, energy efficiency, and carbon footprint.

This review elucidates the electrochemical activity of SiC by integrating experimental findings with theoretical modeling, thereby clarifying how the polytype, nanostructure, and defect chemistry govern lithium storage. It further evaluates sustainable synthesis strategies and binder innovations that couple electrochemical performance to environmental responsibility. By benchmarking bare SiC and heterostructured composites against state-of-the-art anodes, this Review outlines design principles that merge nanoscale engineering with green processing. These insights position SiC not as a passive scaffold but as a viable, partially active host for next-generation, sustainable lithium-ion batteries.

## 2 Structural and Physicochemical Features of SiC

The electrochemical performance of any battery anode is strongly dictated by its intrinsic physicochemical properties and morphological architecture. For SiC, the crystal structure is particularly decisive, as it governs not only the mechanical robustness but also the chemical reactivity, thermal management, and electronic transport properties. These attributes, in turn, define how SiC accommodates lithium storage, tolerates volume fluctuations, and interfaces with the electrolyte. The following sections examine the crystallographic diversity of SiC, its morphological expressions, and the intrinsic properties that collectively underpin its potential as a sustainable anode material.^29^

### 2.1 Crystal Structure of SiC

Silicon and carbon are tetravalent elements with similar electronic structures, possessing only s and p orbitals in their electron clouds.^29^ Consequently, SiC is predominantly covalently bonded, featuring sp^3^-hybridized atoms and a stoichiometric Si:C ratio of 1:1.^30,31^ SiC exhibits polytypism, a phenomenon in which variations in the stacking sequence of its crystal lattice give rise to numerous distinct polymorphic forms. Over 250 SiC polytypes have been identified,^32^ with the cubic, hexagonal, and rhombohedral crystal lattices being the most common.

Multiple notations are used to classify SiC polytypes, including Ramsdell, industrial, ABC, Zhdanov, and Jagodzinski systems. The Ramsdell notation is the most widely used in scientific research. It labels polytypes with a number, indicating the total number of layers in the repeating sequence, followed by a letter that denotes the crystal lattice system (C for cubic, H for hexagonal, and R for rhombohedral). Alternatively, the industrial system categorizes the five most common commercial polytypes into α-SiC and β-SiC, where β-SiC corresponds to the 3C polytype, and α-SiC comprises the noncubic polytypes 2H, 4H, 6H, and 15R.^29^ Figure 2 shows the most common SiC polytypes: (a) 3C, (b) 4H, (c) 6H, and (d) 15R. Specifically, the 3C polytype exhibits a cubic sphalerite-type structure similar to diamond, 4H and 6H polytypes crystallize in a hexagonal wurtzite-type structure, and the 15R polytype displays a rhombohedral or trigonal structure.^30,33^ These structural variations significantly influence the electronic properties of SiC, including its bandgap and charge carrier mobility.^34,35^

**Figure 2:**
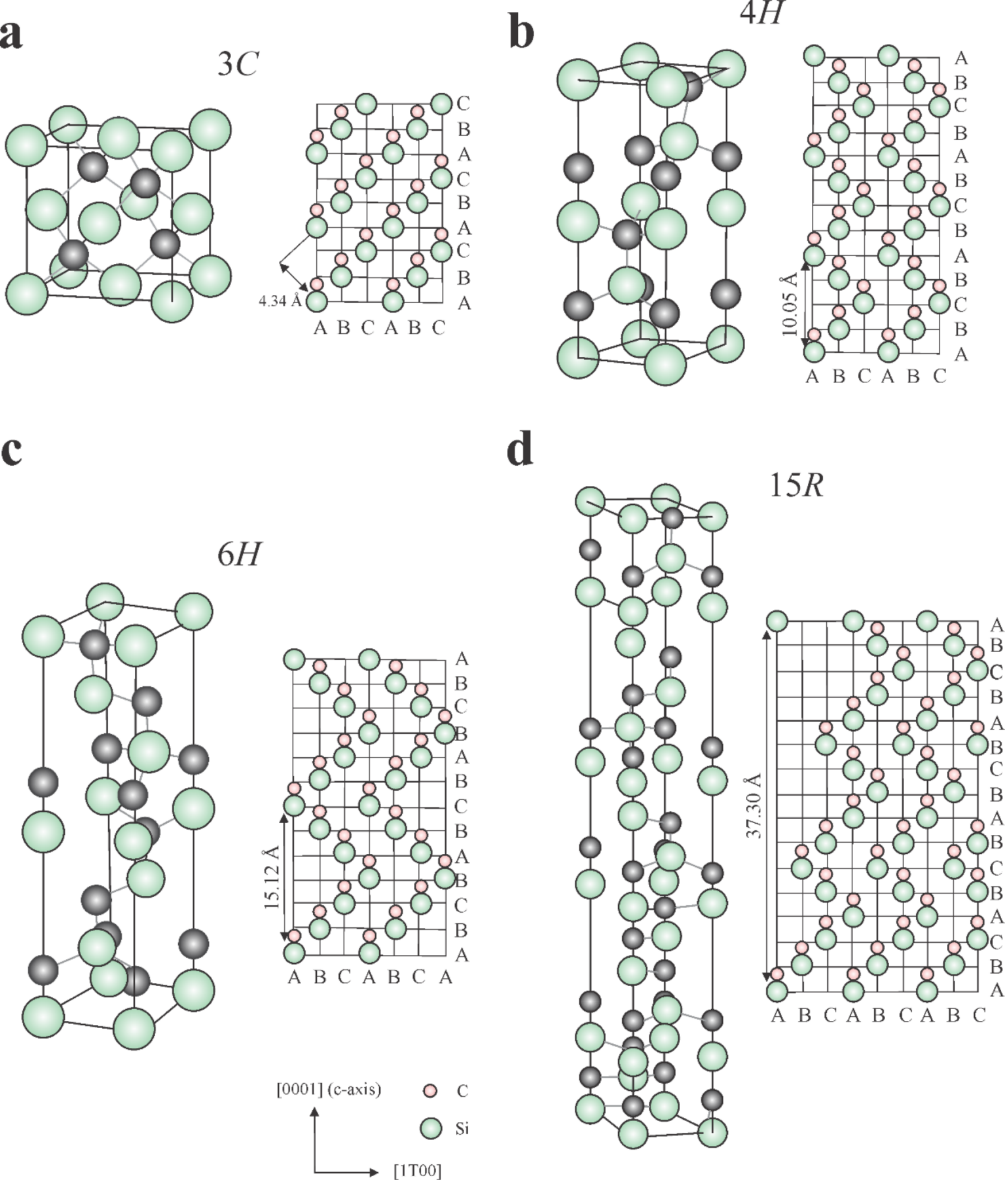
SiC polytypes: (a) 3C (cubic, sphalerite-type), (b) 4H and (c) 6H (hexagonal, wurtzite-type), and (d) 15R (rhombohedral/trigonal).

### 2.2 Morphological Characteristics

Morphology is a critical characteristic that profoundly influences the application and performance of the materials. For instance, Frolova et al. (2023)^36^ studied how variations in SiC particle morphology affect the mechanical characteristics of ceramics, while Wang et al. (2023)^37^ investigated the electromagnetic absorption performance associated with different SiC morphologies. In the context of intercalation batteries, material morphology critically influences the electrochemical behavior and performance, including ion diffusion and interface stability.^8,38,39^ The literature reports a diverse array of SiC morphologies, including nanowires,^40,41^ polygonal granules,^42^ nanorods,^43^ sphere-shaped nanoparticles,^44,45^ lamellar structures,^46^ rod-like forms, and cluster-like or string-beads-like structures.^47^

### 2.3 Intrinsic Properties of SiC Polytypes

SiC is a semiconductor renowned for its exceptional hardness and durability, with a density of about 3.21 g cm^–3^ and high stiffness (Young’s modulus of 390–690 GPa).^34,48^ These mechanical properties are primarily attributed to the strong covalent bonding, high valency, and small atomic radii of silicon and carbon,^29^ making SiC ideal for applications in armor and wear-resistant ceramics. In addition, SiC exhibits excellent thermal management capabilities; for example, its thermal conductivity is 4.9 W cm^–1^ K^–1^ for 6H-SiC, 3.7 W cm^–1^ K^–1^ for 4H-SiC, and 3.2 W cm^–1^ K^–1^ for 3C-SiC, substantially higher than the 1.5 W cm^–1^ K^–1^ for silicon.^49^ Moreover, SiC has a low thermal expansion coefficient of about 4.5 μm K^–1^ m^–1^, which minimizes thermal stress and enhances performance in battery applications.^29^

Table 1 presents a comparative analysis of the electrical properties of silicon and the main SiC polytypes. The SiC bandgap varies by polytype: 3.2 eV for 4H-SiC, 3.0 eV for 6H-SiC, and 2.2 eV for 3C-SiC, all higher than 1.12 eV for Si. This wider bandgap allows SiC devices to operate at higher temperatures and voltages than silicon. Furthermore, SiC has a breakdown field approximately 10 times higher than Si and a saturation drift velocity three times higher, likely due to the stronger Si–C bonding compared to Si–Si bonds.^50^ Although SiC has lower electron mobility than silicon, its high thermal conductivity and superior breakdown electric field suggest that SiC-based devices can achieve higher encapsulation densities and improved current handling capacities.^35^

**Table 1:** Electrical Properties of Si and SiC Polytypes: 3C-SiC, 6H-SiC, and 4H-SiC^a^

**property**	**Si**	**3C-SiC**	**6H-SiC**	**4H-SiC**
bandgap energy (eV)	1.12	2.2	3.0	3.2
breakdown field (MV/cm)	0.3		3.0	3.0
saturation drift velocity (cm/s)	1.0 × 10^–7^	2.7 × 10^–7^	2.0 × 10^–7^	2.7 × 10^–7^
electron mobility (cm^2^/(V·s))	1350	1000	370	980

a The data, sourced from Ruys (2023),^50^ includes key parameters such as bandgap energy, breakdown field, saturation drift velocity, and electron mobility.

Beyond their mechanical and thermal robustness, the different SiC polytypes exhibit distinct electrochemical behaviors that derive from their crystallography and defect chemistry. Nonstoichiometry can drive 3C ↔ 6H transformations, thereby altering electronic states and transport properties in ways that directly affect lithiation pathways.^51^ Recent reports further show that 2H-SiC, although thermodynamically unfavorable for Li insertion in the ideal lattice, becomes electrochemically active in the presence of Si vacancies, which lower insertion energies and enhance conductivity.^52^ First-principles comparisons among 3C, 4H, and 2H in sodium-ion systems indicate that the hexagonal phases (2H, 4H) exhibit lower diffusion barriers and more stable defect formation than cubic 3C.^53^ Extending this concept, layered 2D-SiC architectures have been predicted to deliver reversible capacities approaching 699 mAh g^–1^ with diffusion barriers of ∼0.40 eV, rivaling or surpassing graphite.^54^ These findings reveal that polytype selection is not merely a crystallographic distinction but a determinant of ionic transport and electrochemical activity. Nevertheless, systematic experimental studies directly comparing the performance of 3C, 6H, and 2H under identical electrochemical conditions remain scarce, representing an important knowledge gap for the advancement of SiC-based anodes.

## 3 From Inert Scaffold to the Active Host: A Paradigm Shift in SiC Electrochemistry

SiC has long been regarded as an electrochemically inert scaffold within Si–C composites. Over the past decade, however, this perception has undergone a fundamental transformation. Early studies on nanocrystalline cubic 3C-SiC provided the first compelling evidence of reversible lithium storage, delivering capacities close to 1200 mAh g^–1^ over 200 cycles,^55^ thereby overturning the assumption that SiC merely acted as a mechanically rigid but inactive framework. Amorphous SiC thin films synthesized by ICP-CVD further reinforced this complexity, since they displayed high initial Coulombic efficiencies (ICE) (>80%). At the same time, lithiation proceeded mainly through conversion into Li–Si alloys and Li_*x*_Si_*y*_C phases, which caused severe capacity fading below 400 mAh g^–1^ after 100 cycles.^51^ These contrasting cases established a central principle that persists throughout the literature: morphology dictates the delicate balance between efficiency, stability, and reversibility.

Nanostructuring subsequently emerged as a decisive strategy to overcome the sluggish kinetics and partial irreversibility characteristic of bulk SiC. One-dimensional β-SiC nanowires obtained by molten salt or FFC-Cambridge methods undergo partial decomposition at low potentials, producing Si/Li_*x*_Si_*y*_C domains that subsequently alloy with lithium, while the residual scaffold maintains short diffusion paths and structural integrity. Such architectures have delivered capacities exceeding 700–1000 mAh g^–1^ with near-ideal Coulombic efficiency sustained over hundreds of cycles.^56^ Similarly, twisted SiC nanofibers display progressive electrochemical activation, wherein repeated cycling transforms nominally inert domains into electroactive Si/C composites, stabilizing capacities above 500 mAh g^–1^,^57^ showing how dimensionality, from amorphous thin films to nanowires and nanofibers, directly governs lithiation pathways.

Polytypism adds yet another layer of complexity. While pristine hexagonal 6H-SiC remains largely inactive, surface graphitization under ultrahigh vacuum can activate epitaxial graphene channels, enabling lithium penetration and capacities of ∼670 mAh g^–1^.^58^ At the theoretical level, density functional theory (DFT) consistently predicts that stoichiometric SiC lattices are thermodynamically unfavorable for lithiation, but defect engineering, heteroatom doping, and interfacial coupling make lithium adsorption exothermic and open low-energy diffusion channels.^52,59^ Two-dimensional SiC has been predicted to support a theoretical capacity of ∼699 mAh g^–1^ with migration barriers as low as 0.40 eV,^54^ while van der Waals heterostructures with graphene or borophene may surpass 1900 mAh g^–1^, indicating that interfaces, rather than pristine lattices, will define the next frontier of SiC electrochemistry.^60^

Beyond performance, the emerging literature situates SiC within a broader sustainability framework. Conventional carbothermal synthesis requires extreme temperatures (>1400 °C), and is highly energy-intensive, whereas magnesiothermic reduction enables phase-pure SiC formation at 600–1150 °C and permits the use of renewable precursors such as rice husk, bamboo, or even industrial waste streams.^61,62^ Moreover, unlike pure silicon, which undergoes catastrophic ∼300% volume expansion upon lithiation, SiC-based anodes typically exhibit moderate expansion (∼39%) with reversible recovery, thus harmonizing mechanical stability with electrochemical activity.^63^

These findings delineate a coherent structure-performance framework in which amorphous films offer high ICE but poor retention. Nanowires and nanofibers achieve stable scaffold-assisted alloying; hexagonal polytypes can be activated by interfacial engineering; 3C-SiC uniquely supports reversible solid-solution lithiation; and two-dimensional or heterostructured SiC represents a theoretical frontier for ultrahigh capacity. However, a key unresolved challenge persists in elucidating the relative contributions of decomposition-driven Si formation versus intrinsic intercalation into the SiC lattice. Addressing this question requires a mechanistic lens that combines operando experiments and theoretical modeling to clarify the fundamental lithiation and delithiation pathways of SiC-based anodes.

## 4 Lithiation/Delithiation Mechanisms in SiC-Based Anodes

The recognition of SiC as a partially active host rather than a purely inert scaffold has reshaped research on its lithiation and delithiation behavior. While pristine bulk SiC is thermodynamically unfavorable for lithium insertion, as consistently predicted by DFT (positive lithiation energies across polytypes),^59^ experimental studies reveal measurable capacities once nanoscale architectures, structural defects, or interfacial modifications are introduced.^56,61,64,65^ This contrast reveals a central principle: the activity of SiC is not an intrinsic bulk property but rather emerges from its structural and interfacial engineering.

To understand how these factors govern the electrochemical response, it is useful to classify lithiation and delithiation in SiC into distinct but interrelated pathways. These pathways encompass conversion followed by alloying, nanostructure-enabled processes, and surface and interfacial contributions. Each pathway provides complementary insight into the origin of activity in SiC-based anodes and highlights both opportunities and unresolved challenges.

### 4.1 Conversion versus Alloying Pathways

Amorphous and nanocrystalline SiC films typically undergo an initial conversion reaction, yielding lithium silicon carbide (Li_*x*_Si_*y*_C) and elemental Si, followed by reversible alloying and dealloying of Si with lithium. Broad cathodic signals between 0.5–1.5 V in cyclic voltammetry, and XPS signatures of Li–Si alloys confirm this dual mechanism.^65^ Importantly, residual SiC, although electrochemically inactive, provides a mechanical buffer that mitigates pulverization and strain. In situ stress analyses support this interpretation, showing moderate volume expansion (∼39%) with reversible strain recovery.^63^ Thus, while the conversion–alloying pathway inevitably sacrifices part of the host structure, the structural resilience conferred by unreacted SiC differentiates it from pure Si anodes, highlighting a key advantage of SiC-based systems.

### 4.2 Nanostructure-Enabled Pathways

When engineered at the nanoscale, SiC exhibits lithiation behavior distinct from thin films. β-SiC nanowires, for instance, undergo partial decomposition into Si and Li_*x*_Si_*y*_C at low potentials but retain their scaffold-like integrity, enabling stable alloying reactions with lithium and delivering >700 mAh g^–1^ with high Coulombic efficiency.^56^ Twisted nanofibers display progressive activation, where cycling gradually transforms inert domains into active Si–C composites, stabilizing capacities above 500 mAh g^–1^.^57^ Similarly, porous SiC frameworks obtained by magnesiothermic reduction facilitate ion transport through carbon-coated channels, although their intrinsic lithium diffusivity remains sluggish (∼10^–19^ cm^2^ s^–1^).^61^ These cases reveal that nanostructuring does more than shorten diffusion lengths: it dictates the balance between electrochemical activity and mechanical durability, with architectures that combine short transport pathways and robust scaffolds showing the greatest promise.

### 4.3 Surface and Interfacial Chemistry

Interfacial chemistry may be the most decisive factor in unlocking the SiC activity. Both theory and experiment converge on the observation that lithium preferentially binds to C-terminated SiC surfaces, forming ionic Li–C bonds that lower migration barriers and enable pseudocapacitive contributions.^26,66^ Graphitic coatings or epitaxial graphene layers amplify this effect by stabilizing the SEI and improving charge transfer, in some cases yielding capacities approaching 950 mAh g^–1^ without evidence of crystalline alloy phases.^67^ In heterostructures such as Si@SiC@C, the SiC interlayer further enhances ionic transport while suppressing parasitic reactions with liquid electrolytes, enabling >800 stable cycles with high ICE.^68^ These examples highlight a recurring theme: interfaces in SiC-based anodes are not passive boundaries but active mediators of charge storage and long-term stability.

Mechanistic studies demonstrate that lithiation in SiC is not governed by a single pathway but by a spectrum of interdependent processes including irreversible conversion, reversible alloying, defect-assisted intercalation, and interface-mediated storage. The main unresolved question remains, resolving the relative roles of decomposition-driven Si formation versus intrinsic intercalation into the SiC lattice, as both produce overlapping electrochemical signatures. Addressing this ambiguity will require a coordinated approach that integrates operando spectroscopies, advanced computational modeling, and machine-learning-assisted data interpretation. Only through such an integration can the field establish predictive rules that connect structure, defect chemistry, and interfacial design with the electrochemical performance of SiC-based anodes.

## 5 Theoretical and Computational Insights

Computational studies, particularly those based on DFT, have been pivotal in clarifying the long-standing paradox of SiC: why are pristine crystals electrochemically inert, while modified structures display measurable lithium storage? Across all polytypes, stoichiometric SiC consistently yields positive lithiation energies, making bulk insertion thermodynamically unfavorable.^52,59^ These predictions align with negligible experimental capacities in defect-free lattices and reveal a crucial principle: electrochemical activity in SiC is not intrinsic, but emerges from structural perturbations and interfacial design.

To unravel the atomistic origins of this emergent activity, computational studies have focused on how different structural perturbations influence the lithiation thermodynamics and ion transport. Among these factors, defect and vacancy engineering stand out as the clearest routes to activation, providing both a mechanistic rationale and the most instructive starting point for further discussion.

### 5.1 Defects and Vacancy Engineering

DFT consistently demonstrates that defect chemistry is the most direct route to activating SiC. Introducing silicon vacancies redistributes charge onto neighboring carbon atoms, lowering the bandgap and even inducing metallic conductivity.^52,59^ Migration barriers, prohibitively high in defect-free lattices (≥2 eV), decrease to ∼0.56 eV along carbon-rich channels, consistent with the moderate but reversible storage observed experimentally. These predictions provide a mechanistic rationale for the progressive activation observed in nanofibers and nanowires, where repeated cycling gradually generates defect sites that evolve into lithium hosts. Thus, rather than being purely detrimental, controlled defect creation emerges as a functional design lever.

### 5.2 Doping Strategies and Electronic Structure Modulation

Doping has emerged as a versatile strategy to unlock the electronic potential of SiC, extending the principles of nanoscale activation into deliberate electronic structure engineering. Light-element dopants such as boron and nitrogen exemplify this approach by introducing electron-deficient or -rich sites that stabilize lithium adsorption and enhance conductivity. Computational and experimental studies on nanoribbon and nanocrystalline SiC have demonstrated that lithiation can induce semiconductor-to-metal transitions in these doped systems, directly supporting fast charge transport and improved rate performance.^69^ Moreover, DFT-predicted codoping schemes suggest synergistic effects, simultaneously lowering diffusion barriers and increasing theoretical capacities, although these predictions remain to be validated experimentally. These findings emphasize that the rational selection of dopants not only influences the thermodynamics of lithium storage but also determines whether SiC operates as a sluggish semiconductor or as a quasi-metallic host.

Beyond light elements, transition-metal incorporation provides an additional pathway to modulate the electronic landscape. Jiang et al. (2024)^27^ showed that Zr-doped polymer-derived SiC undergoes a sequential phase evolution from amorphous ZrO_2_ to t-ZrO_2_ and ultimately to ZrC, accompanied by the redistribution of free-carbon ribbons that form extended conductive networks. This synergistic interplay between dopant chemistry and carbon organization dramatically reduced the bandgap from ∼3.2 to ∼1 eV and raised electrical conductivity to 0.28 S cm^–1^, as supported by DFT calculations. Importantly, these materials delivered stable cycling and high Coulombic efficiency when tested as LIB anodes, providing direct evidence that dopant-driven electronic modulation translated into electrochemical benefits. These advances demonstrate that doping in SiC is far more than a substitutional tactic, functioning instead as a multiscale design pathway in which light-element dopants tailor electronic states to promote lithiation kinetics, while transition-metal dopants couple phase evolution with carbon-mediated conductivity. In both cases, doping redefines the way SiC accommodates charge transport and stabilizes interfacial reactions, positioning electronic structure modulation as a critical frontier for the advancement of SiC-based anodes.

### 5.3 Dimensionality and Nanoscale Architectures

DFT also reveals how dimensionality fundamentally redefines lithiation pathways. Two-dimensional SiC sheets are predicted to host capacities near 699 mAh g^–1^ with migration barriers as low as 0.40 eV,^54^ nearly double that of graphite. Defective nanotubes present quasi-one-dimensional diffusion channels with ultralow barriers (∼0.10 eV),^70^ suggesting exceptional rate capability. Nanowires and bilayer models exhibit semiconductor-to-metal transitions during lithiation, echoing experimentally observed progressive activation in fibrous and wire-like morphologies.^71,72^ These insights highlight that nanoscale architectures confer both electronic flexibility and structural adaptability absent in bulk crystals, bridging theoretical predictions with experimental outcomes.

### 5.4 Interfacial Architectures and Heterostructures

Perhaps the most transformative computational predictions involve interfaces. van der Waals heterostructures between SiC and conductive 2D materials (borophene, graphene, and BN) generate internal electric fields that redistribute charge and dramatically reduce diffusion barriers. SiC/borophene, for instance, supports multilayer lithium adsorption with theoretical capacities above 1900 mAh g^–1^, while SiC/graphene and SiC/BN reduce migration barriers to ∼0.55 eV and enhance structural resilience.^60,73,74^ These predictions rationalize experimental observations of high capacities in epitaxially graphitized polytypes^58^ and in Si@SiC@C heterostructures,^68^ illustrating how atomic-scale modeling directly informs device-level performance.

Theoretical and computational studies converge on three recurring principles: defects stabilize lithium binding and open diffusion channels; dopants and heterostructures redistribute charge and enhance conductivity; and nanoscale dimensionality enables lithiation with limited volume change. These insights form a predictive framework capable of reconciling experimental controversies, particularly the debate between decomposition-driven alloying and intrinsic intercalation. Moving forward, coupling *ab initio* predictions with operando diagnostics and machine-learning-assisted screening will be essential to accelerate the rational design of SiC anodes. Computational insights thus move beyond abstract predictions, defining the structural and electronic motifs that synthesis must target for SiC to mature into a practical anode for next-generation lithium-ion batteries.

## 6 Synthesis of SiC from Sustainable Precursors

SiC has long captivated researchers, not only as an advanced ceramic material but also as stardust observed around carbon-rich stars. Naturally occurring SiC, known as moissanite, was first observed by Ferdinand Henri Moissan in a meteorite in Arizona in 1893.^75^ Although SiC is rare on Earth, large-scale production was pioneered in the late 19th century with the Acheson method, patented by Edward Goodrich Acheson in 1893, a process that remains widely used today.^75–77^ The Acheson method employs carbothermic reduction, in which a silicon source is reduced by a carbon source (eq 1) at temperatures above 2000 °C in an electrically heated resistance furnace.^78^

1 $${\mathrm{SiO}}_{2}+3C\to \mathrm{SiC}+2\mathrm{CO}$$

The synthesis of SiC is governed by a complex series of solid–solid, solid–gas, and gas–gas reactions,^79^ as can be seen in eqs 2 to 7:

2 $${\mathrm{SiO}}_{2}+C\to \mathrm{SiO}+\mathrm{CO}$$

3 SiO+C→Si+CO

4 Si+C→SiC

5 $${\mathrm{SiO}}_{2}+\mathrm{CO}\to \mathrm{SiO}+{\mathrm{CO}}_{2}$$

6 SiO+2C→SiC+CO

7 $$2\mathrm{SiO}\to \mathrm{Si}+{\mathrm{SiO}}_{2}$$

Currently, studies have focused on exploring alternative raw materials for SiC synthesis and promoting more sustainable and environmentally friendly production methods. Table 2 shows the reaction temperatures, synthesis method, and crystallite sizes obtained when using green feedstocks, such as wood, recyclables, and agricultural residue. These feedstocks are abundant, inexpensive, and inherently carbon-rich, making them attractive for sustainable SiC production.

**Table 2:** Synthesis of SiC from Wood, Recyclables, and Agricultural Residue Materials, Temperature, and Method of Preparation, and Crystallite Size and Type

**raw material**	**temperature** **(°C)**	**preparation method**	**crystallite size and type**	**references**
rice husk and magnesium powder	650–1150	magnesiothermic reduction	SiC samples have a cubic phase and the samples synthesized at 950 °C have a cubic phase and a small amount of hexagonal phase	^90^
sawdust of Peruvian wood	1500	carbothermic reaction		^91^
barley husks, barley husk fly ash, and pyrolyzed barley husks	160 (air) and 700–100 (nitrogen)	carbothermic reaction - magnesiothermic reduction	crystallites cubic phase 5.32, 14.2, and 5.43 nm and crystallite hexagonal phase 14.3, 8.3, and 13.24 nm of barley husks, barley husk fly ash, and pyrolyzed barley husks, respectively	^92^
glasses and postconsumer coffee grounds	1150–1450	carbothermic reaction	nanowire diameters of 50–100 nm	^40^
sludge waste powders and petroleum coke	1750	carbothermic reaction	samples are cubic SiC as the main phase with 1–10 μm regular and well-defined crystalline particles	^42^
rice husk	1000–1500	carbothermic reaction	samples were a mixture of particles and whiskers	^93^
wood (unbleached and bleached pulp)	1400	carbothermic reaction	the unbleached samples are 5–25 μm in diameter and up to a few cm in length, and the bleached samples are 250 nm in diameter and 5.0 mm in length, both with cubic SiC	^43^
rice husk, coconut shell, and poly carbosilane	1400	carbothermic reaction	SiC nanowire structures with diameters of 20 – 150 nm and lengths up to several microns. SiC without poly carbosilane are carbon phases cubic SiC, α-cristobalite, and graphite. SiC whisker growth was enhanced with the addition of poly carbosilane	^41^
waste tire rubber shreds and glass chunks (computer monitors and TV screens)	1550	carbothermic reaction	the samples are nanofibers of 5 μm in length and 10–150 nm diameter and nanoparticles of 30–40 nm. the SiC samples have cubic and hexagonal phases	^94^
bamboo leaves and tetraethyl orthosilicate	1300–1400	carbothermic reaction	hexagonal SiC nanowires have a diameter of 60–160 nm and lengths up to tens of microns. cubic SiC is found on the joints in branched and in the nodes of the bamboo-like nanowires	^95^
macadamia shell waste and silica	1550	carbothermic reaction	cubic SiC is sphere-shaped nanoparticles with a diameter of 20–80 nm	^44^
ecklonia radiata macroalga	1200–1800	carbothermic reaction	the samples have nanowires (length 10 μm with diameter of 70–100 nm) and lamellar structures (up to 2 μm length and 13 nm thicknesses). predominantly cubic phase on nanowires and hexagonal phase on nanorods	^46^
coal and sandstone	1300–1600	carbothermic reaction	cubic SiC	^96^
printed circuit boards and compact disks	1350	carbothermic reaction	cubic SiC particles are spherical with a diameter of 20–100 nm	^45^
bamboo	1500–1700	carbothermic reaction	samples are cubic SiC and a trace amount of hexagonal SiC	^97^
rice husk and magnesium powder	600	magnesiothermic reduction	cubic SiC has a uniform size of 20–30 nm	^80^
barley husks and magnesium powder	reactor with resistance-heated tungsten wire at a 5 A current	magnesiothermic reduction	cubic SiC with size of 4–6 nm and hexagonal SiC with size of 14–20 nm	^98^
rice husks	1300–1800	carbothermic reaction	the samples are cubic SiC with a residual amount of hexagonal SiC in the form of whiskers and/or particles.	^99^
bamboo slices	1300	carbothermic reaction	SiC nanowires are cubic phase with a diameter of 20–70 nm and a length of 10–20 μm	^100^
bamboo charcoal	1400–1800	carbothermic reaction	cubic SiC with different shapes rod-like and granular was obtained. gray-green SiC powders are expected to be a new raw material	^47^
palm kernel shell	1400	carbothermic reaction	SiC nanowhiskers are cubic phase	^101^
rattan	1500	carbothermic reaction	rod-like SiC whisker and tablet-like SiC particle with cubic phase	^102^
wood-fruit	1400	carbothermic reaction	ultralong SiC nanowires with cubic phase and a residual amount of hexagonal	^103^
cornstalk	1200–1400	carbothermic reaction	cubic SiC nanowhiskers with a diameter of 100 nm and length of 5 mm	^104^

### 6.1 Carbothermic Reduction from Biomass and Recyclables

Carbothermal processing remains the most widely employed route, with synthesis temperatures typically ranging from 1200 to 1800 °C. Depending on precursor composition and reaction conditions, the resulting SiC encompasses a broad spectrum of morphologies from nanowhiskers and nanowires to micrometer-sized particles with cubic and hexagonal phases or both (Table 2). For instance, glass and coffee-ground mixtures yielded highly crystalline SiC nanowires of 50–100 nm diameter at 1450 °C,^40^ while sludge waste powders and petroleum coke produced cubic SiC with well-defined crystallites of 1–10 μm at 1750 °C.^42^ Agricultural residues consistently form whisker-like morphologies, with cubic SiC dominating and residual hexagonal SiC, as can be seen in Table 2. These examples illustrate that precursor chemistry strongly influences microstructure and phase purity, with lignocellulosic feedstocks typically favoring whisker growth and silicate-rich precursors enabling particulate morphologies.

Although these approaches make effective use of renewable or waste feedstocks, their sustainability is tempered by the substantial energy input required to sustain reaction temperatures of 1500–1800 °C. From a rigorous green perspective, the environmental benefit of biomass-derived carbothermal synthesis cannot be judged solely on precursor origin but must also consider life-cycle and energy-return-on-investment (EROEI) metrics. Such analyses remain largely absent from the current literature. As a result, while carbothermal methods demonstrate versatility and scalability, their true sustainability profile remains uncertain. Alternative strategies, such as magnesiothermic or sol–gel reductions, molten-salt processes with recyclability, or even solar-assisted carbothermal systems, are therefore especially attractive, as they offer pathways to reduce energy intensity while retaining structural control.

### 6.2 Magnesiothermic Reduction and Molten-Salt-Assisted Routes

Magnesiothermic reduction offers a lower-temperature alternative, enabling SiC formation between 600 and 1150 °C, as can be seen in Table 2. In this process, magnesium acts as a reducing agent, as described in eq 8, although the precise mechanisms and intermediate species remain under debate. Studies on rice husk-derived silica demonstrated that magnesiothermic reduction yields phase-pure cubic SiC with uniform particle sizes of 20–30 nm at 600 °C, albeit with modest yields of 7–10 wt %.^80^ An often-overlooked aspect of this route is the formation of MgO as a byproduct. Although MgO can be readily removed by acid leaching,^80^ this step generates liquid effluents that compromise the overall sustainability of the process. In principle, MgO could also be recycled or repurposed as a flux or precursor in other processes, which would help reduce waste generation and improve the environmental balance of this reaction. MgO has been applied in various areas, such as high-performance refractories,^81^ pharmaceutical industry,^82,83^ pollutant removal,^84–87^ catalyst (sunflower oil transesterification),^88^ and energy storage.^89^

8 $${\mathrm{SiO}}_{2}+2\mathrm{Mg}+C\to \mathrm{SiC}+2\mathrm{MgO}$$

Molten-salt-assisted reactions further lower kinetic barriers, with intermediates such as Mg_2_Si facilitating SiC formation through multistep pathways described through the eqs 9, 10, 11, 12, and 13.^105^ These approaches produce SiC with controlled size distributions, high purity, and tunable morphologies while dramatically reducing energy input compared with conventional carbothermal routes.

9 $${\mathrm{SiO}}_{2}+4\mathrm{Mg}\to {\mathrm{Mg}}_{2}\mathrm{Si}+2\mathrm{MgO}$$

10 $${\mathrm{Mg}}_{2}\mathrm{Si}+{\mathrm{SiO}}_{2}\to 2\mathrm{Si}+2\mathrm{MgO}$$

11 $${\mathrm{SiO}}_{2}+2\mathrm{Mg}\to \mathrm{Si}+2\mathrm{MgO}$$

12 Si+C→SiC

13 $${\mathrm{SiO}}_{2}+{\mathrm{Mg}}_{2}\mathrm{Si}+2C\to 2\mathrm{SiC}+2\mathrm{MgO}$$

### 6.3 Molten-Salt Electrolysis of Biomass Precursors

A more recent advance leverages molten-salt electrolysis to directly convert rice husks (RHs) into SiC or Si–C composites.^106^ Figure 3 shows an illustration of SiC and C–Si obtained from rice husks via molten-salt electrolysis. RHs are particularly attractive, as they naturally accumulate silica and carbon during growth, functioning as a sustainable dual-source precursor. In molten CaCl_2_, RH-derived SiO_2_–C mixtures are electrochemically reduced to C-SiC composites containing SiC nanowires (<10 nm) that deliver >1000 mAh g^–1^ at 1 A g^–1^ over 400 cycles. In contrast, molten NaCl-KCl-MgCl_2_ yields C–Si composites with capacities of 926 mAh g^–1^ at 500 mA g^–1^ after 100 cycles. This comparison highlights that although both products originate from the same biomass precursor, SiC-based composites exhibit higher stability and longer cycle life than Si-based analogues, underlining the SiC advantage as a green anode material. Thus, rice husk valorization not only illustrates the feasibility of electrochemical green synthesis as a complement to thermal reduction but also provides a direct benchmark where SiC outperforms other sustainable anodes derived from the same resource.

**Figure 3:**
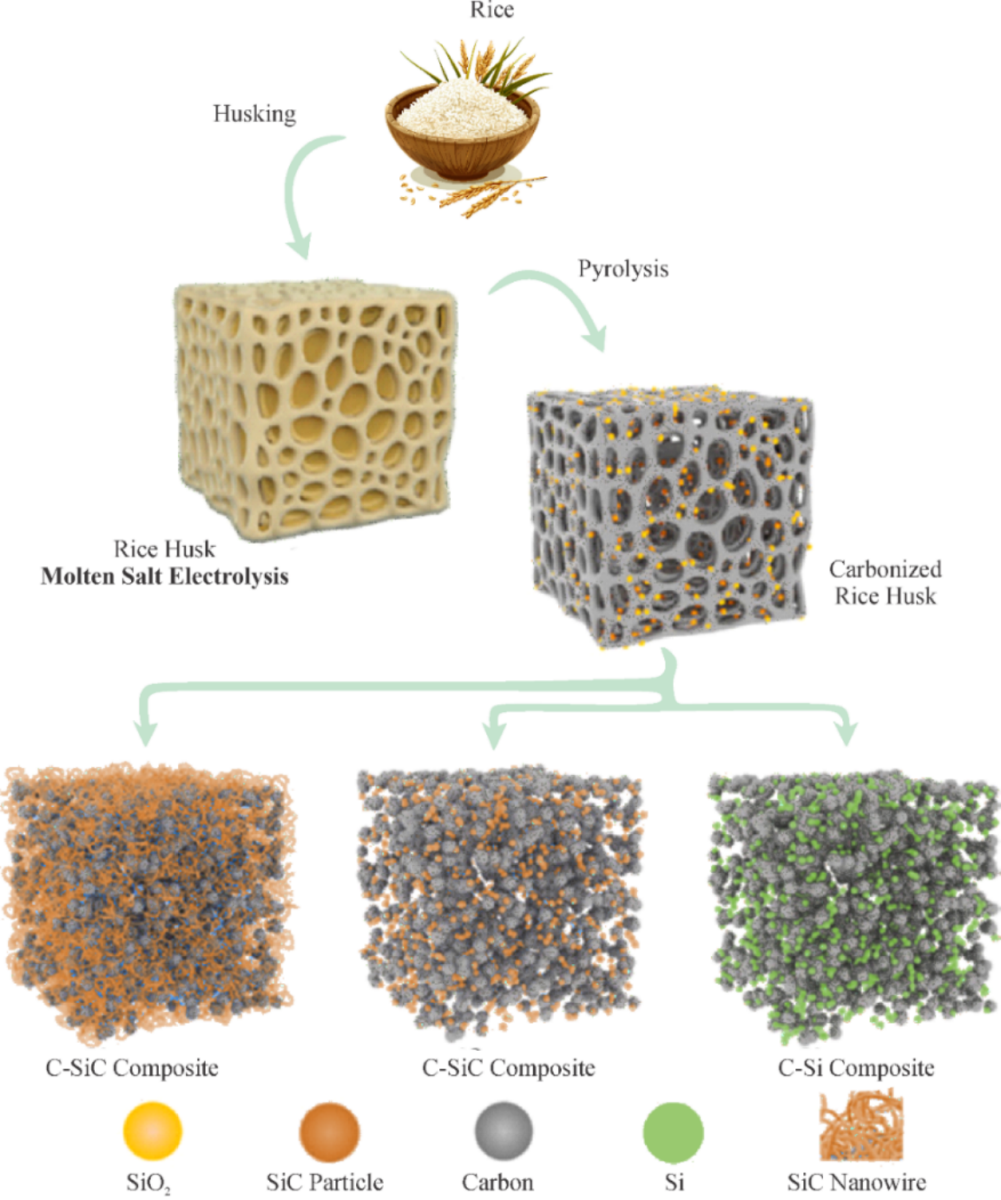
Schematic illustration of SiC and C–Si synthesis from rice husks by a molten-salt electrolysis approach. Adapted from Zhao et al. (2019).^106^

### 6.4 Advanced Low-Carbon Processing

Beyond precursor selection, innovative processing technologies are emerging to further minimize the carbon footprint of the SiC synthesis. A notable example is the use of concentrated solar furnaces, where tetraethyl orthosilicate and sucrose precursors were converted to SiC at 1500 °C with significantly reduced CO_2_ emissions compared to conventional furnaces.^107^ This illustrates how coupling renewable feedstocks with sustainable energy sources can address both the material and process dimensions of green synthesis.

From a battery perspective, sustainable synthesis must be assessed not only in terms of environmental benefits but also by how it governs the structure and performance of SiC. Biomass-derived precursors provide compelling examples of this interplay, as rice husk, barley husk, and resin–silica sources have been transformed into SiC nanofibers, spheres, and nanotubes, architectures that translate into higher initial efficiencies and improved capacity retention.^57,108^ In contrast, conventional silica-derived SiC often exhibits poor reversibility and limited ICE, as illustrated by silica-gel-based SiC spheres.^109^ These cases highlight how green feedstocks are not passive substitutes but active design elements that couple sustainability to the nanostructuring pathways that underpin battery performance.

## 7 Binder Chemistry in SiC-C Anodes

Binders are far more than inert additives in composite electrodes; they dictate structural integrity, adhesion to the current collector, and preservation of electronic pathways during lithiation-delithiation. Conventional systems rely almost exclusively on PVDF processed in *N*-methyl-2-pyrrolidone (NMP), which is valued for chemical stability and ease of slurry casting.^56,57,108,110–117^ However, the intrinsic limitations of this platform are increasingly evident. The nonpolar backbone of PVDF restricts peel strength and elasticity, while the reliance on toxic, expensive solvents raises environmental and economic concerns. Moreover, PVDF often fails at the electrode–electrolyte interface, especially in Si-rich electrodes where HF evolves from LiPF_6_ decomposition, leading to poor SEI stability and rapid capacity fading.^118,119^ These shortcomings elevate binder chemistry from a processing detail to a design variable central to the performance of SiC anodes. Several solvents have been highlighted in the literature as a green alternative for PVDF processing, including triacetin, triethylene glycol diacetate (TEGDA), ethylene carbonate, dimethyl sulfoxide (DMSO), acetyl tributyl citrate (ATBC), triethyl phosphate (TEP), methyl 4-dimethylcarbamoyl-2-methylbutanoate, dihydrolevoglucosenone, and N, N′-dialkyldibutylsuccindiamides.^120^

As illustrated in Figure 4, bioderived binders address precisely these limitations by coupling sustainability with interfacial functionality. Derived from abundant, renewable feedstocks, such as cellulose, alginate, and starch, aqueous binders eliminate the hazards of NMP while introducing polar functional groups that promote multipoint anchoring, ionic coordination, and tunable mechanical resilience. The advantages extend beyond sustainability: water solubility, low cost, and versatility in hydrogen-bonding chemistry collectively redefine the role of the binder in SiC electrodes.

**Figure 4:**
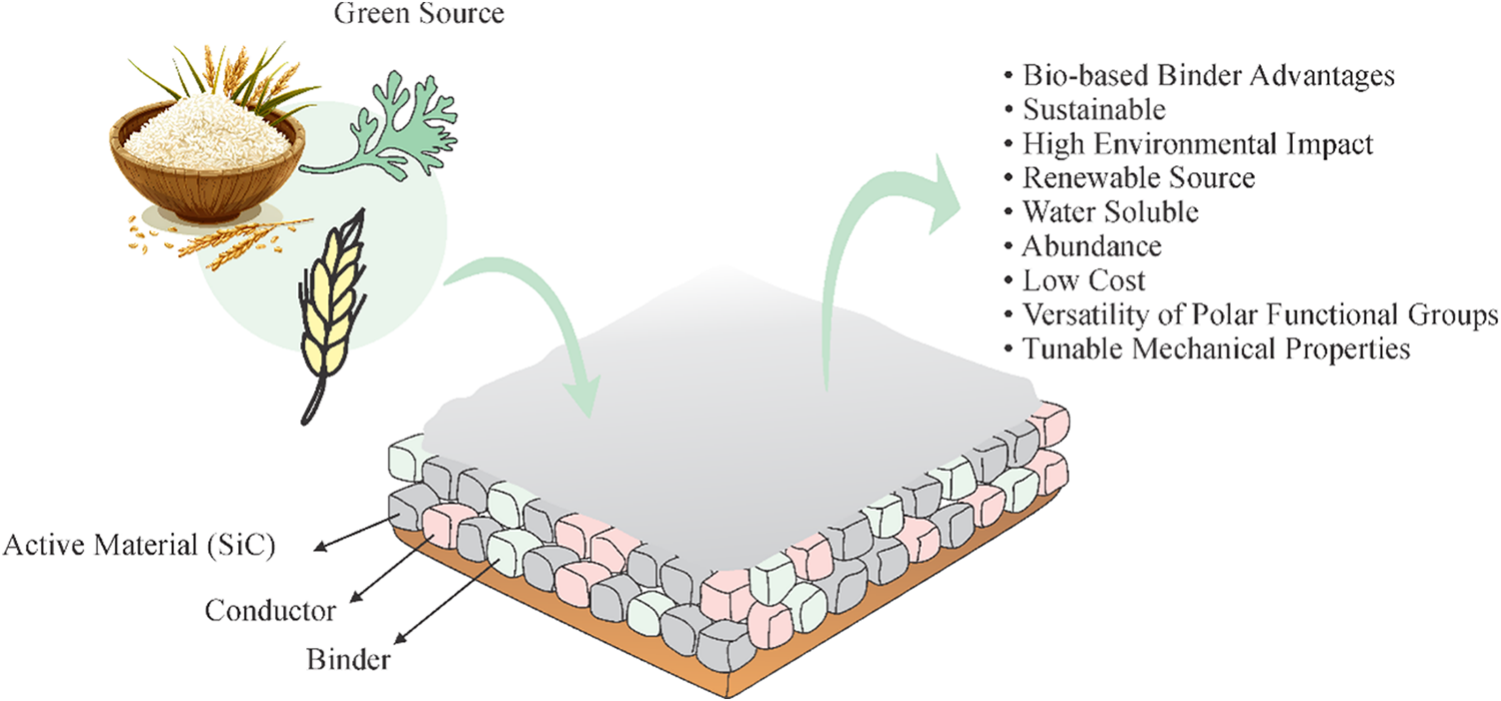
Representative schematic of a LIB electrode showing its main components, highlighting the binders, specifically those from natural sources.

More transformative progress arises from aqueous, biobased binders such as carboxymethyl cellulose (CMC), poly­(acrylic acid) (PAA), and alginate. Their polar groups form dense hydrogen-bonding and ionic coordination networks with oxidized SiC surfaces, thereby reinforcing cohesion and suppressing the growth of unstable SEI.^121–126^ CMC, often blended with styrene–butadiene rubber (SBR), has become the benchmark for this class, where ionic anchoring and elastic recovery jointly mitigate cracking and preserve conductive pathways.^122^ PAA and lithium polyacrylate offer comparable adhesion, while functionalized derivatives, such as dopamine-modified CMC or NaPAA-*g*-CMC, further enhance ICE and long-term stability by suppressing parasitic reactions.^127,128^ Alginate, with its uniformly distributed −COOH groups, provides particularly strong multipoint anchoring and ionic cross-linking, translating directly into stable SEI formation and improved retention.^122^

Beyond naturally derived polymers, synthetic aqueous binders broaden the design landscape. Polyimides (PI) exhibit excellent elasticity, bonding strength, and thermal stability, often outperforming PVDF in Si-based systems.^129–132^ Their limitations remain largely economic, with most commercial formulations still reliant on NMP, although water-processable versions are emerging. Acrylonitrile copolymers such as LA133 represent a pragmatic option, especially when paired with electrolyte additives like fluoroethylene carbonate or LiPO_2_F_2_, which collectively improve SiC electrode stability.^133^ Other process-driven approaches, while promising, remain constrained. Solvent-free PTFE processing eliminates drying and solvent hazards but continues to face dispersion and adhesion issues.^118,134^ Other process-centric strategies, such as solvent-free PTFE processing or binder-free nanowire electrodes, offer niche advantages but face scalability and cost barriers.^135,136^ The recent advances in some representative binders and ink specifications are summarized in Table 3.

**Table 3:** Active Material, Binder, Conductor, and Solvent for the LIBs Ink Preparation

**active material**	**binder**	**conductor**	**proportion active (wt %) active material: conductive agent: binder**	**solvent**	**references**
SiC/C composites (SiC from barley husks and graphite)	CMC and SBR	carbon black	80:10:10 (CMC:SBR, 1:1)	water	^98^
SiO2@C or p-SiOx/SiC@C	SA	carbon black	80:10:10 (2% aqueous solution)	water	^138^
pSi/SiC	SA	carbon black super P	60:20:20	water	^137^
C/SiC/Si	SA	acetylene black	63.75:21.15:15	water	^139^
Si/SiC	SA	carbon black	70:15:15	water	^105^
C–Si alloys	PI	carbon black	62.5:18.5:19	NMP	^129^
Gr–SiC–Si NWs	PI	carbon black	80:10:10	NMP	^130^
SiC particles	PVDF	carbon black	80:10:10	NMP	^63^
Si/SiC	SA	carbon black	70:15:15	water	^105^
CNT/SiNPs, CNT/SiNPs/AC or CNT/SiNPs/SiC	CMC	carbon black	60:20:20	water	^140^
Si/SiC/C	CMC	acetylene black	90:5:5	water	^141^
Si@SiC@PF nanocomposite	CMC	carbon black	70:20:10	water	^142^
crystalline Si and SiC	CMC	carbon black	70:10:20	water	^109^
CNT/SiNPs/SiC	CMC	carbon black	60:20:20	water	^140^
nano SiC	CMC	carbon black	65:20:15	water	^55^
Fe2O3@SiC nanowires	CMC	carbon black	80:10:10	water	^143^
SiC composite (waste silicon)	PVDF	acetylene black	80:10:10	NMP	^144^
Si-SiC@C (rice husk)	CMC and SBR	carbon black	60:20:20	water	^3^
β SiC/Si	CMC and SBR	carbon black	80:10:10 (CMC 5 wt % and SBR 5 wt %)		^145^
Si-nano, Si-macro, and SiC	PVDF	carbon black	80:15:5	NMP	^62^
SiC powders	PVDF	carbon black	80:15:5	NMP	^61^
SiC powders	PVDF	carbon black	80:10:10	NMP	^56^
SiC powders	PVDF	acetylene black	80:10:10	NMP	^57^
SiC/C composite mesoporous nanotubes	PVDF	acetylene black	75:12.5:12.5	NMP	^108^
SiC@C nanocomposite	PVDF	acetylene black	80:10:10	NMP	^110^
nc-Si/SiC composite	PVDF	acetylene black	40:40:20	NMP	^111^
Si/SiC/C in situ composite microspindles	PVDF	carbon black	80:10:10	NMP	^114^
Si/SiC/nanographite	PVDF	acetylene black	80:10:10	NMP	^112^
S-carbon nanotubes/SiC	PVDF	acetylene black	80:10:10	NMP	^113^
Si/SiC/C nanocomposite microspheres	PVDF	acetylene black	80:10:10	NMP	^114^
SiC@HGSs	PVDF	acetylene black	70:20:10	NMP	^117^
carbon polyhedron@SiC@Si	PVDF	superconductor black	80:10:10	NMP	^115^
SiC-produced and commercial SiC β-type	Na-CMC	carbon black	65:20:15	water	^55^
recycled Si/SiC composite	SA	carbon black	75:10:15	water	^146^
SiC@SiO_2_–CSNWs nanowires	no binder	graphite paper		solvent free	^135^
SiC@silicon core–shell nanowires	no binder	carbon paper		solvent free	^136^
Si/SiC/CNT nanocomposite	polyacrylic acid	carbon black	80:10:10	water	^147^
Si@SiC	lithium polyacrylate	carbon black	70:20:10		^148^
commercial SiC600	acrylonitrile multicopolymer	carbon black	80:10:10	water	^133^
crystalline silicon and SiC	Na-CMC	carbon black	70:10:20	water	^109^

The comparative performance of these systems is displayed in Figure 5, which shows the trade-off between ICE and long-term retention in SiC-based anodes. In Figure 5a, PVDF/NMP systems (orange) typically exhibit higher ICE (>70%) but lower capacity retention, consistent with their limited mechanical compliance and unstable SEI formation. In contrast, aqueous binders (green) display wider variability in ICE, with some systems initiating below 40%, but they deliver markedly superior retention, often above 90%, highlighting the stabilizing role of polar functional groups. Figure 5b consolidates these findings at the category level, showing that PVDF/NMP binders average 67.9% ICE and 73.2% retention, while aqueous systems average 58.8% ICE but achieve an impressive 91.1% retention. This dichotomy highlights a mechanistic shift: conventional binders prioritize initial reversibility, whereas aqueous binders foster long-term resilience. From a practical standpoint, aqueous systems, despite their ICE penalty, are attractive for full-cell integration, where prelithiation strategies can offset first-cycle inefficiencies. These insights establish binder chemistry not as an auxiliary consideration but as a central design lever for advancing sustainable, high-performance SiC anodes.

**Figure 5:**
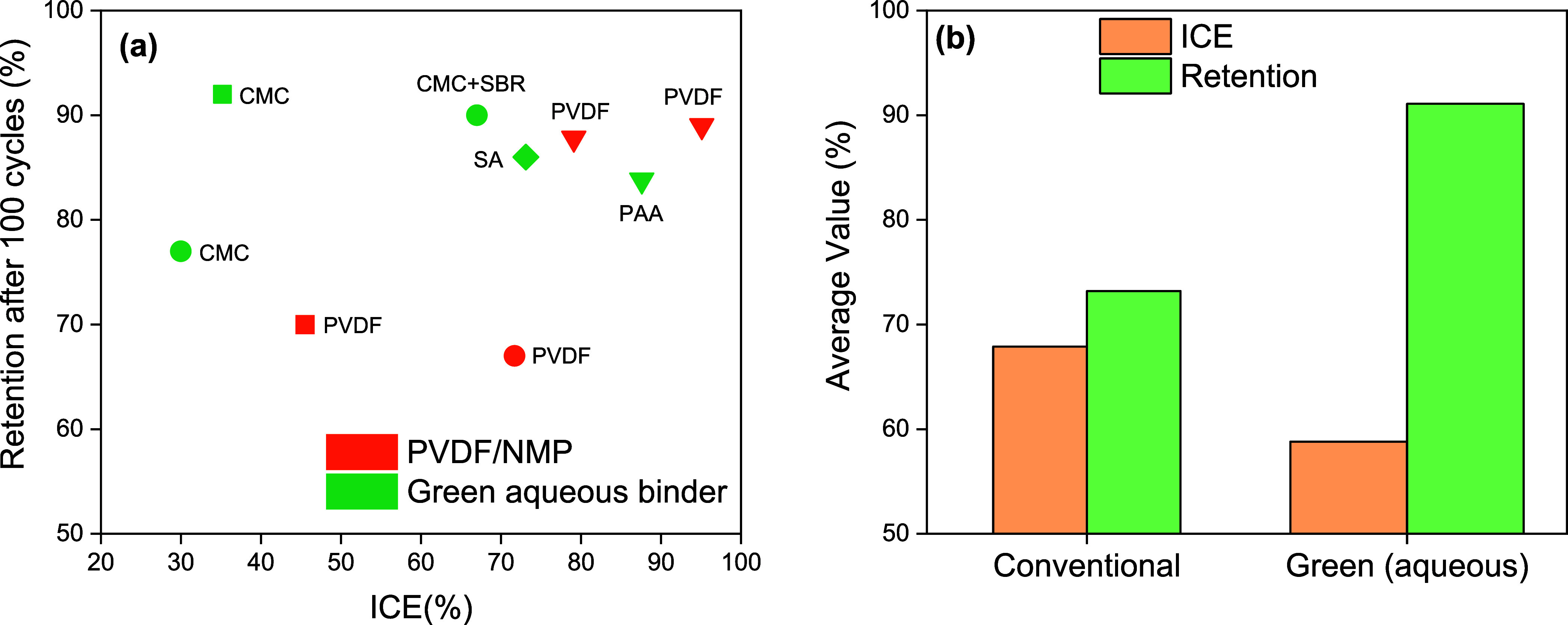
(a) Capacity retention after 100 cycles versus ICE. Symbol shapes denote active materials. ■ SiC,^55,56^ ● SiC–C,^57,98,109^ ◆ Si/SiC,^137^ ▼ Si/SiC/C.^114^ (b) Average ICE and capacity retention after 100 cycles for conventional (PVDF/NMP) and aqueous binders.

## 8 From Bare SiC to Heterostructured Architectures

The recognition of SiC as an electrochemically active phase emerged first from studies of bare SiC, where the absence of conductive buffers made mechanistic signatures more transparent. Early demonstrations with nanocrystalline 3C-SiC synthesized by CVD challenged the long-standing view of SiC as inert, delivering ≈1200 mAh g^–1^ over 200 cycles, well above graphite (372 mAh g^–1^), with remarkable rate resilience and full recovery after high-rate cycling.^55^ These results pointed to surface-driven processes, including SEI formation and partial graphitization, as key activation pathways.

Morphology further dictated performance. β-SiC nanowires displayed capacities of 2266/1031 mAh g^–1^ (charge/discharge) at 0.5 A g^–1^, retaining ∼720 mAh g^–1^ after 100 cycles with rapid stabilization of Coulombic efficiency ∼99.4% and a low-voltage plateau consistent with alloying-like reactions.^56^ In contrast, bulk defect-poor 6H-SiC wafers were nearly inert until activated by annealing, which induced nitrogen doping and surface graphitization; the transformation yielded areal capacities of 34–41 mAh cm^–2^ (vs 0.6 mAh cm^–2^ in pristine wafers), corresponding to ∼670 mAh g^–1^.^58^ Amorphous thin films added further nuance, as a 250 nm film reached 1427 mAh g^–1^ initially but suffered severe fading, while a thicker 500 nm film offered a lower initial capacity of 917 mAh g^–1^ but improved retention because residual SiC buffered stress during alloying cycles.^65^

Theory provides a unifying rationale for these observations. DFT calculations confirm that perfect bulk SiC resists lithiation thermodynamically, whereas silicon vacancies, boron doping, and surface reconstructions lower insertion energies and diffusion barriers.^59,69^ Ultrathin SiC slabs spontaneously reconstruct into graphitic-like layers, favoring interlayer lithiation at ∼0.20 V, consistent with graphitization-driven activity in nanocrystalline SiC.^52^ Extending this, two-dimensional SiC layers are predicted to deliver 699 mAh g^–1^ with diffusion barriers of ∼0.40 eV, approaching or surpassing graphite bilayers.^54^ These results demonstrate that the activity of SiC is not intrinsic but emerges from nanoscale perturbations, graphitic reconstructions, and interfacial stabilization.

Building on this mechanistic foundation, research progressed toward SiC-C heterostructures, where carbon frameworks amplify and stabilize the intrinsic contributions of SiC. In contrast to Si-rich ternary Si-SiC-C systems, where alloying of Si dominates, binary SiC-C composites allow a clearer evaluation of the SiC role. Their preparation includes carbothermal, magnesiothermic, coating/decomposition, and molten-salt electrolysis methods. Representative synthesis routes, together with their practical trade-offs, are summarized in Table 4. Carbothermal reduction remains the most established but requires ≥1400 °C, raising scalability and sustainability concerns.^57,64^ Magnesiothermic routes reduce the thermal budget and enable biomass-derived precursors, but MgO removal and byproduct management complicate scale-up.^98,149^ Coating and decomposition strategies, such as conformal carbon shells or polydopamine-derived coatings, stabilize SEI and improve conductivity but introduce cost and complexity.^28,150,151^ More recently, molten-salt electrolysis has emerged as a disruptive alternative, producing tunable nanostructures at intermediate temperatures (∼850 °C) with potentially lower environmental impact.^106^

**Table 4:** Summary of Reported Synthesis Routes for the SiC-C Composites

**synthesis method**	**conditions**	**category**	**references**
carbothermal reduction of RF-SiO_2_ nanofibers + HF etching	1400 °C, Ar	carbothermal	^57^
PMAA@SiO_2_@RF templating + carbothermal reduction	1600 °C, Ar	carbothermal/hybrid	^117^
carbothermal synthesis (SiO_2_ + C → C/SiC) + HF treatment	>1400 °C, Ar	carbothermal	^64^
modified Stöber → RF carbon coating → magnesiothermic reduction (Mg, 800 °C)	800 °C, Mg, Ar	magnesiothermic	^152^
in situ carbothermic-magnesiothermic reduction	800–1000 °C, Mg + C	magnesiothermic hybrid	^62^
in situ thermochemical method using soda papermaking black liquor	800 °C, reductive organics	magnesiothermic	^149^
magnesiothermic reduction of nSiO_2_ (barley husks) + induction annealing (2400 °C)	800 + 2400 °C, Mg + Ar	magnesiothermic	^98^
ball-milling slurry casting + double-wrapping binder coating		coating/decomposition	^150^
HF treatment + polydopamine coating + pyrolysis	800 °C, 2 h, Ar	coating/decomposition	^151^
direct thermal decomposition (epitaxial NG growth in NH_3_)	1500 °C, NH_3_	coating/decompositin	^28^
molten-salt electrolysis (CaCl_2_, 2.4–2.8 V, ∼850 °C)	850 °C, CaCl_2_ molten	molten salt	^106^

Mechanistically, these heterostructures fall into three regimes. Table 5 summarizes representative examples classified into Regimes A–C, which provide a conceptual framework linking synthesis to the electrochemical response. Regime A (SiC-active) involves ordered SiC-C junctions and graphitic coatings that reduce Li^+^-diffusion barriers and enable intercalation-like storage without alloying plateaus.^117,152,153^ Regime B (partial conversion/Si-alloying) arises when ultrafine domains or defects release electroactive Si, yielding capacity activation but broader hysteresis and strong dependence on SEI stabilization.^57,62^ Regime C (carbon-dominated) describes systems where SiC acts mainly as a template or catalyst for graphitization, while storage occurs predominantly in carbon.^98,149^ Importantly, regime assignment is not intrinsic to SiC but strongly conditioned by the synthesis route and microstructural context: carbothermal/decomposition products align with Regime A, magnesiothermic hybrids often resemble Regime B, and coating/template strategies tend toward Regime C.

**Table 5:** Mechanisms Identified in SiC–C Composites^a^

**material**	**mechanistic regime** **(A/B/C)**	**lithium-storage mechanism (type)**	**key evidence**	**references**
EG@SiC (epitaxial graphene-coated SiC)	A - SiC-active	Li_*x*_SiC, solid-solution/intercalation	no Si-plateau; Schottky junction lowers Li^+^ barrier 0.74→0.37 eV; stable SEI; high-rate retention	^153^
SCS/SiC@SiC/C hollow nanospheres	A - SiC-active	Li_*x*_SiC, solid-solution/intercalation	SEI humps (∼1.0/0.7 V) then stabilize; capacity activation with cycling; carbon shell aids transport	^152^
bowl-like 3C-SiC nanoshells in hollow graphitic spheres (SiC@HGSs)	A - SiC-active	Li_*x*_SiC, solid-solution/intercalation	reversible lithiation; graphitic shells shorten Li^+^ paths; surface graphitization; durable rates	^117^
NG@SiC (N-doped graphene on SiC)	A - SiC-active	Li_*x*_SiC, solid-solution/intercalation	C–Si–C bridges; Li^+^ barrier 0.84→0.43 eV; DFT support; no alloying plateaus	^28^
C-SiC composite (SiC nanowires <10 nm + amorphous C, molten CaCl_2_)	A - SiC-active	Li_*x*_SiC, solid-solution/intercalation	no plateau <0.25 V; coulombic efficiency ∼99%; gradual capacity activation; intimate SiC-C framework	^106^
SiCA@C (HF + polydopamine coating + pyrolysis)	supportive (leans A)	mixed (moderate SiC-active + capacitive)	SiO_*x*_ removal; N-doped C boosts Li^+^ diffusion (∼35×); robust high-rate cycling	^151^
SiC1000@LiPAA/3D-s-PU (SiC + SWCNT/LiPAA + PU)	supportive (A/B)	mixed (SiC-active + capacitive)	swelling reduced; D_Li^+^_ ∼ 45–66 × 10^–6^ cm^2^ s^–1^; durable SEI; improved kinetics	^150^
twisted SiC-C nanofibers (92.5 wt % β-SiC)	B - conversion/Si-alloying	Si-alloying (conversion via Si)	SiC→Si + C upon cycling; capacity growth; scaffold prevents pulverization; sloping/polypeak profiles	^57^
SiC-C (nanocomposite, ∼45% C)	mixed (tends B)	Si-alloying + partial reversible Li-SiC	high first discharge; D_Li^+^_ ∼1.09 × 10^–9^ cm^2^ s^–1^; *R*_ct_ ∼ 145 Ω; stable carbonic SEI	^62^
SiC-C (70.5SiC–29.5C, wt %)	mixed (tends C)	graphitic-C intercalation (LiC_6_) + slow SiC participation + pseudocapacitive	peaks <0.22 and ∼0.05 V; Coulombic efficiency → ∼99% after formation; graphite-dominated storage	^64^
graphene/SiC nanosheets (∼8:1)	C - template/scaffold	template/scaffold (active = graphitic-C)	porous nanosheets shorten paths; performance dominated by graphene; improved stability	^149^
GC-1.4 (graphitic carbon from nSiC/C precursor)	C - template → graphitization	template/scaffold → graphitic-C intercalation (LiC_6_)	SiC drives graphitization; LiC_6_ intercalation in graphitic domains	^98^

a Notes. A = SiC-active (lithium storage via solid-solution/intercalation into SiC, i.e., Li_*x*_SiC); B = partial conversion/Si-alloying (capacity dominated by Li–Si alloy formation with SiC acting primarily as a rigid scaffold); C = template/scaffold (performance governed by graphitic carbon; SiC directs graphitization or provides mechanical reinforcement). “Mixed/Supportive” entries are assigned to the regime that best reflects the predominant behavior.

Despite this framework, key ambiguities persist. Overlapping electrochemical signatures make it difficult to distinguish Regime A from Regime B, as sloping voltage profiles or subtle low-voltage features may reflect either intercalation-like or conversion pathways. Quantitative partitioning of capacity among SiC, residual Si, and carbon remains seldom resolved, and extrinsic factors such as additives, binder chemistry, and electrode porosity further confound attribution.^150^ Reports of “capacity activation” remain mechanistically ambiguous, possibly reflecting not only Li_*x*_SiC percolation but also SEI densification, stress-induced defects, or electrolyte wetting effects.^3,61,63^

The progression from bare SiC to SiC-C heterostructures demonstrates that the electrochemical activity in SiC is not intrinsic but engineered. Bare systems illuminate the fundamental levers, nanosizing, amorphization, defects, and graphitization, while heterostructures translate these into practical designs that stabilize transport, buffer volume change, and sustain cycling. Mechanistic regimes (A–C) provide a useful conceptual map but remain blurred by overlapping signatures and extrinsic variables. Resolving these ambiguities requires coordinated *operando* spectroscopy, systematic defect quantification, and integration with DFT and machine learning, enabling predictive design of SiC-based anodes for sustainable lithium-ion batteries.

### 8.1 Performance Hierarchy and Trade-Offs

Figures 6 and 7 delineate the initial performance hierarchy of SiC-C anodes by mapping the ICE and first-cycle charge capacity across representative architectures. A clear stratification emerges in Figure 6, where electrodes designed with rational interfacial or electronic engineering, such as epitaxial or graphene-derived coatings on SiC (EG@SiC, SiCA@C),^151,153^ and binder-optimized systems like SiC1000@LiPAA/3D-PU,^150^ consistently achieve efficiencies in the range of 82–95%. In contrast, conventional bulk composites occupy a broader and lower efficiency window of 31–67%,^64,98,106,152^ while near-pure SiC nanofibers stabilize around ∼72% ICE.^57^ This ranking demonstrates that first-cycle irreversibility is dictated primarily by interfacial chemistry and electronic connectivity at the SiC/carbon/electrolyte junction rather than by the overall carbon fraction.

**Figure 6:**
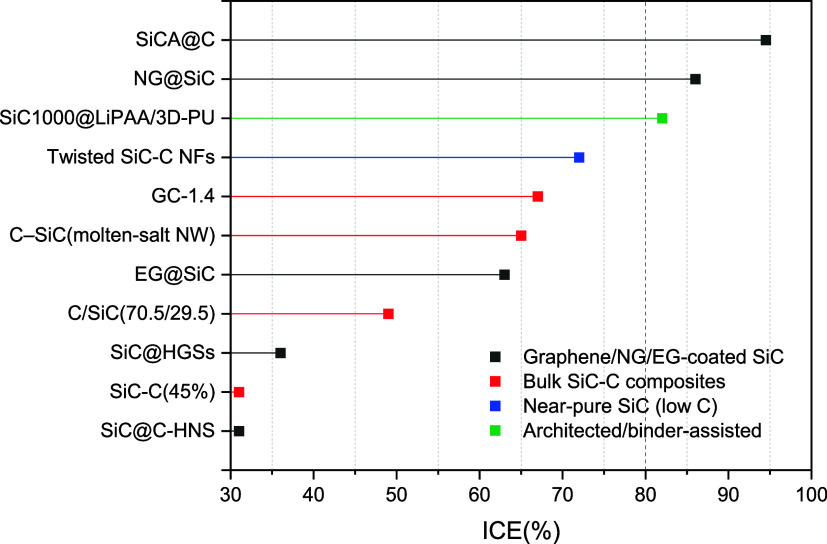
ICE of SiC-C composites by material and architecture.

**Figure 7:**
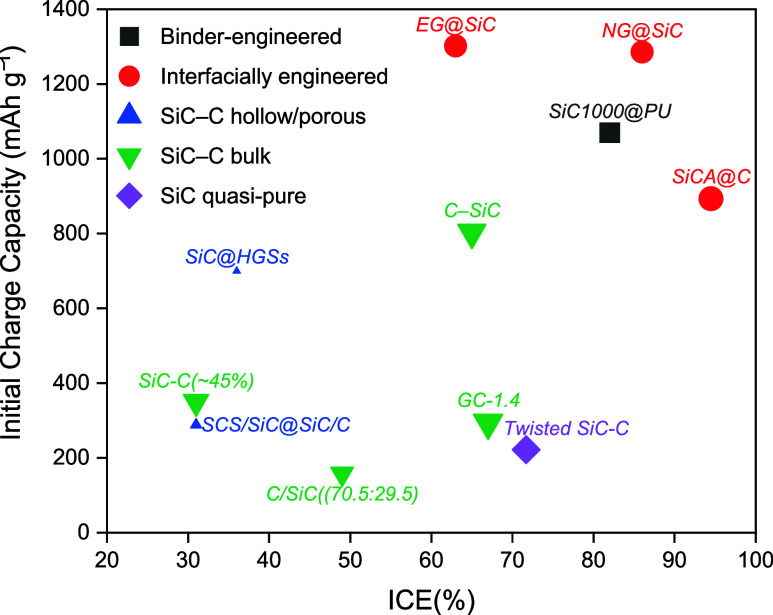
Initial charge capacity vs ICE for SiC-C anodes. Larger symbols indicate data collected at lower current densities; initial capacity values were measured at 0.1 A g^–1^, while higher currents of 0.5 and 0.6 A g^–1^ were used for SCS/SiC@SiC/C and SiC@HGSs, respectively. This scaling enables a direct comparison of performance trends across diverse testing conditions.

When capacity is plotted against ICE (Figure 7), the central trade-off becomes explicit. The highest capacities, approaching 1000–1300 mAh g^–1^ for EG@SiC and NG@SiC,^28,153^ are obtained at the expense of moderate efficiencies (∼85–90%). By contrast, near-pure SiC nanofibers exhibit respectable ICE (∼72%) but limited reversible capacity (∼220 mAh g^–1^),^57^ reflecting conversion-limited activation with restricted generation of electroactive Si. Hollow and porous composites, such as SiC@HGSs and SCS/SiC@SiC/C, occupy an intermediate regime with capacities of 400–700 mAh g^–1^ but suffer from very low ICE values (∼31–36%).^117,152^ These behaviors are consistent with extensive electrolyte penetration into defect-rich domains and uncontrolled SEI growth, leading to substantial irreversible lithium consumption. These data highlight the central dilemma of the SiC-C heterostructures: no single architecture simultaneously maximizes ICE and reversible capacity, and performance gains are achieved only through deliberate compromise.

The mechanistic origins of this hierarchy suggest that the superior ICE of coated and binder-engineered electrodes arises from reduced electronic tortuosity, improved current percolation, and the suppression of parasitic electrolyte reduction. Continuous sp^2^ shells in EG@SiC, NG@SiC, and SiCA@C provide uniform, thin SEI films that minimize lithium consumption during formation.^28,151,153^ Binder architectures offer a complementary route, as SiC1000@LiPAA/3D-PU reaches ∼1070 mAh g^–1^ at ∼82% ICE,^150^ demonstrating how Li-carboxylate binders reinforce interparticle contacts, buffer mechanical stress, and chemically direct SEI formation. In contrast, the low-ICE tail observed in bulk and hollow composites reflects chemically unstable surfaces and excessive reactive area, where interfacial losses dominate despite higher theoretical capacities.

A closer look reveals that ICE is governed primarily by interfacial chemistry and mechanical stability, while the capacity depends on the density of accessible active domains and the continuity of electronic percolation. Improving one axis without sacrificing the other requires co-optimization: conductive and chemically benign carbon shells to provide electronic pathways and passivation, multifunctional binders that serve simultaneously as mechanical buffers and interphase reagents, and controlled porosity that facilitates ion transport without amplifying first-cycle consumption. Simply increasing free carbon or surface area elevates capacity at the expense of ICE, while suppressing reactivity without restoring percolation limits the achievable capacity. The outperforming electrodes succeed precisely because they integrate these design levers, sp^2^ networks, interfacial passivation, and structural compliance, to shift toward both higher ICE and higher reversible capacity.

Nevertheless, two limitations constrain direct cross-study comparison. First, ICE is highly sensitive to formation conditions, including electrolyte formulation (FEC content), voltage cutoffs, current density, areal loading, and the use or omission of prelithiation, which can significantly alter measured values. Second, several reports present only first-cycle discharge capacity, obscuring the true ICE. For decision-useful reporting, studies should consistently provide both charge and discharge capacities, ICE, current density, areal loading, voltage window, electrolyte/additives, and at least one interfacial probe, such as XPS depth profiling or in situ EIS during formation, to allow meaningful comparison across architectures. In this context, Figures 6 and 7 frame not only the hierarchy of initial performance but also the methodological discipline required to assess SiC-C electrodes with rigor.

### 8.2 Rate Capability and Cycling Stability

Figure 8 plots the capacity of representative SiC–C anodes after 100 cycles as a function of current density and demonstrates that long-term performance is dictated more by electrode architecture than by current density alone. Interfacially engineered systems (EG@SiC, SiCA@C, NG@SiC) consistently outperform the graphite benchmark (∼372 mAh g^–1^), sustaining ∼900–1250 mAh g^–1^ at 0.1 A g^–1^.^28,151,153^ Binder-engineered SiC (SiC1000@LiPAA/3D-PU) likewise occupies the high-capacity regime at moderate current densities, showing the ability of mechanically compliant, ion-conductive binders to stabilize particle-binder-collector contacts and mitigate SEI fracture.^150^ Hollow and porous composites (SiC@HGSs, SCS/SiC@SiC/C) achieve intermediate values (∼500–700 mAh g^–1^ at 0.5–0.6 A g^–1^), consistent with fast ion transport through open textures but penalized by large reactive areas that promote parasitic electrolyte reduction.^117,152^ Bulk SiC-C composites cluster near the graphite line, reflecting their sensitivity to carbon fraction, connectivity, and defect chemistry.^64^ In contrast, quasipure SiC nanofibers remain below graphite even at low current densities, consistent with conversion-limited activation and the limited generation of electroactive Si.^57^ Despite variations in test protocols, these architectural trends consistently emerge, indicating that rational interfacial design and binder engineering expand the current-capacity envelope by stabilizing charge transport and mitigating parasitic reactions, whereas unpassivated porosity and quasipure SiC remain fundamentally limited by interfacial instabilities that promote irreversible losses.

**Figure 8:**
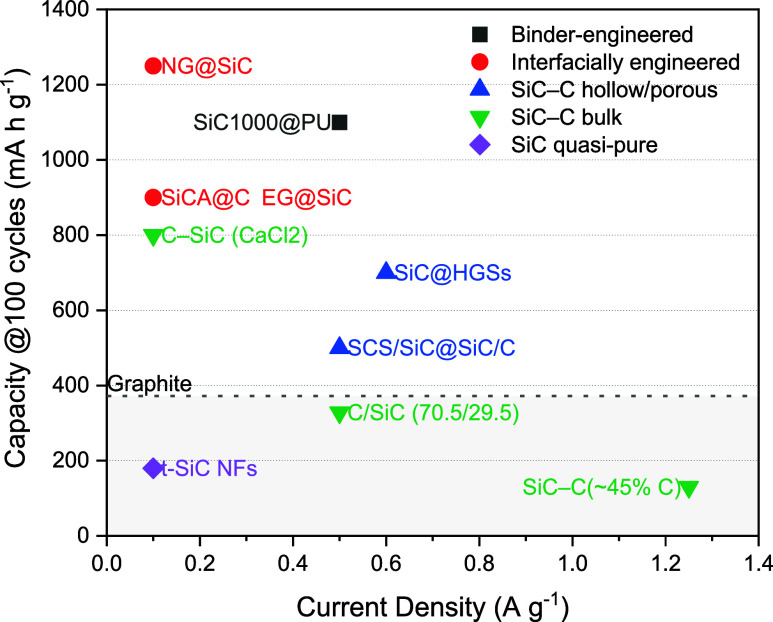
Capacity after 100 cycles vs current density for representative SiC-C electrodes.

Rate capability profiles (Figure 9) provide further evidence of this hierarchy. When normalized to initial capacity, interfacially engineered electrodes (NG@SiC, EG@SiC, SiCA@C) retain ∼60–70% at 2 A g^–1^,^28,151,153^ demonstrating the effectiveness of conformal sp^2^ shells in suppressing polarization and stabilizing the SEI under fast cycling. Binder-engineered SiC (SiC1000@LiPAA/3D-PU) shows comparable retention, confirming that multifunctional binders can serve as an alternative to interfacial coatings for maintaining electrode integrity under high-rate operation.^150^ In contrast, hollow and porous composites (SiC@HGSs, SCS/SiC@SiC/C)^117,152^ and bulk SiC-C materials (GC-1.4, C-SiC, SiC-45%C, G/SiC-NS)^62,98,106,149^ degrade more steeply, typically retaining only ∼40–55% of capacity at 2 A g^–1^. The steepest decline is observed in quasipure SiC nanofibers (<40%),^57^ reflecting the intrinsic limitations of conversion-dominated lithiation in the absence of engineered interfaces.

**Figure 9:**
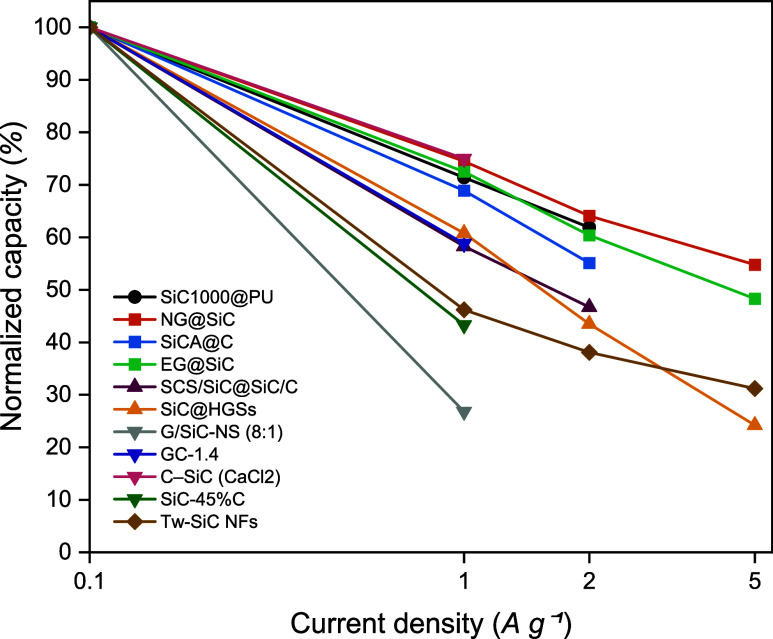
Normalized rate capability of representative SiC-C anodes plotted as a function of current density (0.1–5 A g^–1^, log scale). Data are normalized to the capacity at 0.1 A g^–1^ for each sample. Symbols indicate the architectural class: circles = binder engineering (SiC1000@PU), squares = interfacial engineering (NG@SiC, SiCA@C, and EG@SiC), upward triangles = hollow/porous (SCS/SiC@SiC/C, and SiC@HGSs), downward triangles = bulk (G/SiC-NS, GC-1.4, C-SiC, and SiC-45%C), and diamonds = SiC quasipure (Tw-SiC NFs).

The mechanistic picture remains consistent across both data sets, showing that ICE and long-term stability are governed by interfacial chemistry, whereas high-rate performance depends on the balance between electronic percolation and controlled ion pathways. Electrodes that integrate continuous sp^2^ shells, interfacial passivation, and compliant binder networks succeed in combining high efficiency, rate capability, and cycling stability, while designs that rely solely on high surface area or excess free carbon elevate the initial capacity but suffer from accelerated degradation under stress conditions.

Thus, Figures 8 and 9 establish a performance hierarchy for the rate capability and cycling stability: Interfacial ≈ Binder > Hollow/Porous > Bulk > Quasipure. This ordering shows that durable high-rate SiC-C anodes cannot be achieved by compositional tuning alone but require deliberate integration of interfacial chemistry, electronic connectivity, and mechanical compliance. These design principles provide a roadmap for translating early cycle advantages into long-term, practical performance.

### 8.3 Synthesis Routes to Performance and Sustainability

Figure 10 correlates the synthesis route of SiC-C anodes with their electrochemical outcomes, revealing a hierarchy shaped by both the interfacial stability and the sustainability of the underlying chemistry. Carbothermal reduction delivers moderate ICE (∼54%) and competitive reversible capacity (∼572 mAh g^–1^ at 1 A g^–1^), but the extreme energy demand of high-temperature processing (>1400 °C) raises concerns for low-carbon, large-scale deployment despite structural robustness.^57,117^ Magnesiothermic reduction, although operating at a lower temperature, combines poor ICE (∼43%) with limited capacity (∼217 mAh g^–1^) and generates MgO-rich byproducts that complicate purification and increase waste burdens.^62,98,152^ Molten salt-mediated syntheses achieve somewhat higher ICE (∼65%) and intermediate capacity (∼450 mAh g^–1^), but their reliance on large salt fluxes raises questions of recyclability and environmental footprint despite enabling lower processing temperatures.^106^ By contrast, CVD and coating-based strategies provide the most balanced outcomes, with high ICE (∼80%) and superior capacity (∼729 mAh g^–1^) through deliberate interfacial engineering.^28,150,151^ Nevertheless, their dependence on precursor gases, vacuum infrastructure, and energy-intensive equipment limits their scalability and undermines their environmental credentials, even as they set the current benchmark in performance.

**Figure 10:**
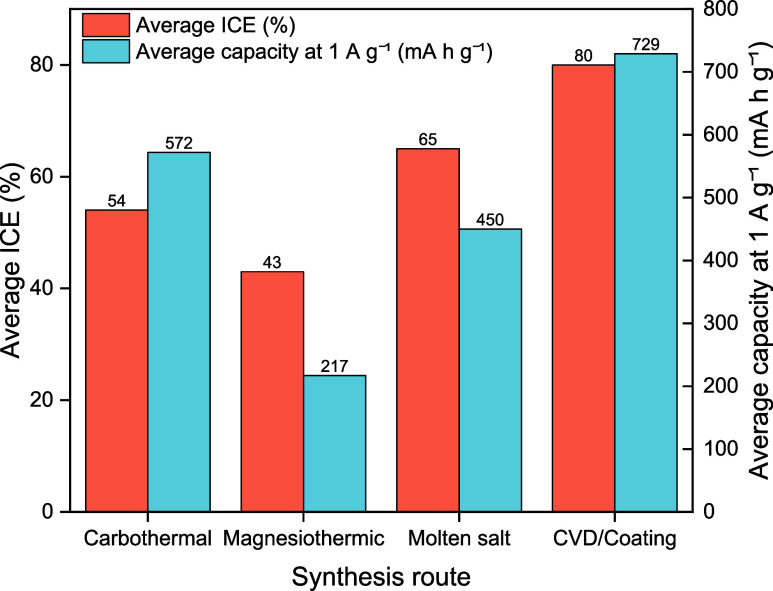
Comparison of average ICE (left axis) and average reversible capacity at 1 A g^–1^ (right axis) of SiC-based anodes synthesized by different routes. Bars are color-coded by metric: orange for ICE and blue for capacity.

These comparisons emphasize a dual imperative of maximizing the electrochemical efficiency while simultaneously minimizing the energetic and chemical footprint of synthesis. Conventional carbothermal and molten-salt routes illustrate the trade-off between scalability and sustainability; magnesiothermic processes highlight the risks of uncontrolled reactivity; and CVD-derived coatings demonstrate the power, but also the cost, of interfacial engineering. Moving forward, greener adaptations such as biomass-derived carbothermal silica, recyclable molten-salt media, low-temperature sol–gel chemistry, or bioinspired coatings will be essential to reconcile high performance with environmental responsibility. This synthesis–performance map, therefore, crystallizes the overarching message of this review: the future of SiC-C anodes hinges not only on breaking the ICE-capacity trade-off but also on embedding interfacial engineering within sustainable process design. Advances in efficiency and capacity will translate to viable next-generation lithium-ion batteries only if achieved through pathways that are scalable, low-carbon, and environmentally responsible.

From an industrial perspective, the viability of SiC-based anodes depends equally on their economic and environmental feasibility. Conventional carbothermal reduction, historically employed for bulk SiC production, operates at 1400–1800 °C with long dwell times and carbonaceous reductants, resulting in high energy consumption and substantial CO_2_ emissions.^64,154^ Although mature and reliable for refractory applications, such old-fashioned processes are increasingly incompatible with modern low-carbon manufacturing goals. In contrast, low-temperature and sustainable synthesis approaches, notably magnesiothermic reduction and molten-salt electrolysis, enable SiC formation at 600–1150 °C and ∼850 °C, respectively, significantly reducing energy intensity and permitting near-carbon-neutral operation when coupled with renewable heat sources.^106,152^

Although direct techno-economic data for SiC anodes are still unavailable, analogies with related systems provide useful insights. Graphite anodes cost about 4 to 10 dollars per kilogram, reflecting process maturity and resource abundance.^155,156^ In contrast, porous silicon made through magnesiothermic reduction costs more, ranging from 40 to 75 dollars per kilogram, due to limitations in magnesium recovery and batch scalability.^157,158^ Considering its lower reaction temperature, compatibility with renewable feedstocks, and structural robustness, SiC produced through magnesiothermic reduction or molten-salt electrolysis likely falls within an intermediate cost range, positioned above graphite but potentially below silicon. These sustainable processes thus bridge electrochemical performance and manufacturability, indicating that future techno-economic and life-cycle assessments will be crucial for quantifying their advantages. Including these factors expands the role of SiC from a purely electrochemical material to a model for the sustainable process design in next-generation batteries. The combination of moderate energy density, long cycle life, and environmentally responsible synthesis demonstrates that performance improvements must go hand-in-hand with scalable, low-carbon manufacturing methods.

Positioning SiC within the broader context of next-generation lithium-ion anodes reveals its distinctive balance among capacity, structural stability, and sustainability. Whereas alloy-type anodes such as Si, Sn, and Ge achieve high theoretical capacities (3579–4200 mAh g^–1^), their extreme volume expansion (>250%) during lithiation induces mechanical degradation, loss of electrical contact, and unstable SEI growth, leading to rapid capacity fading and safety risks.^159–163^ In contrast, SiC exhibits a limited volume expansion, maintaining electrode integrity through the robustness of the Si–C covalent framework, which resists pulverization and minimizes irreversible reactions.^57,68,164^ Electrochemically, SiC operates through a hybrid conversion-alloying mechanism in which partial reduction of SiC yields a small fraction of active Si that participates reversibly in Li–Si alloy formation. The residual SiC matrix functions as a mechanical and electronic backbone, providing confinement and conductivity during cycling. This mechanism explains the high-capacity retention (>90% after hundreds of cycles) and thermal stability observed in SiC-C composites, attributes rarely achieved by pure Si, Sn, or Ge anodes.^65,109^

Compared with hard carbon, SiC presents a complementary profile. Hard carbons offer superior rate capability and lower working potential, but their limited mechanical resilience and structural disorder can compromise long-term stability.^165^ On the other hand, SiC provides exceptional mechanical integrity, chemical inertness, and tolerance to high temperatures, making it particularly attractive for high-safety applications such as electric vehicles and stationary storage.^65^ Similarly, while lithium metal remains the ultimate high-capacity benchmark (3860 mAh g^–1^), its dendrite formation and severe reactivity pose safety and manufacturing challenges.^166–169^ SiC inert surface chemistry naturally suppresses dendrite growth and prevents thermal runaway, offering a safer compromise with moderate but durable capacity.^57^

From a sustainable perspective, SiC also stands out. Unlike alloy-type or metallic anodes that rely on energy-intensive metallurgical processing, SiC can be produced from biogenic SiO_2_–C precursors (rice husks, bamboo, sawdust) through low-temperature magnesiothermic reduction or molten-salt routes, drastically reducing energy demand and CO_2_ emissions compared with conventional carbothermal or CVD-based synthesis.^109^ These green approaches not only lower the environmental burden but also align with circular economy principles by valorizing agricultural waste and enabling reagent recovery.

Finally, SiC represents a good balance between performance and sustainability. Although its gravimetric capacity remains lower than that of Si, Sn, and Li, SiC offers superior mechanical robustness, enhanced safety, and greater environmental compatibility. Ongoing advances in defect engineering, heteroatom doping, and hybrid composite design have positioned SiC as a mechanically resilient and chemically stable framework for sustainable, next-generation lithium-ion batteries, capable of combining high electrochemical performance with low-carbon manufacturing pathways.

## 9 Conclusions and Outlook

SiC is a conditionally active anode material whose electrochemical contribution emerges only when nanosizing, defect engineering, and carbon integration are deliberately orchestrated. Experimental evidence and theoretical modeling converge on the message that SiC activity is not intrinsic but is unlocked through tailored lattice energetics and interfacial chemistry that stabilizes Li–C interactions and lowers migration barriers. Nevertheless, the origin of reversible capacity, whether from decomposition-driven Si formation, intrinsic SiC activity, or carbon contributions, remains unresolved due to overlapping signatures, making rigorous operando studies and quantitative partitioning essential. Looking forward, three complementary frontiers define the path ahead: first, multimodal operando diagnostics integrated with predictive simulations are needed to clarify lithiation pathways; second, machine-learning-assisted computation can accelerate the screening of defect chemistries, dopants, and interfacial motifs to enable predictive design; third, sustainable processing must extend beyond microstructural precision to embrace the full electrode architecture, including low-carbon synthesis routes, green binders, aqueous processing, and biomass-derived or recyclable precursors. If these integrative strategies succeed, SiC could transition from a mechanistic curiosity to a sustainable and scalable anode candidate, reshaping not only the performance portfolio of next-generation lithium-ion batteries but also their environmental footprint.
